# Cannabidiol (CBD) modulation of apelin in acute respiratory distress syndrome

**DOI:** 10.1111/jcmm.15883

**Published:** 2020-10-15

**Authors:** Évila Lopes Salles, Hesam Khodadadi, Abbas Jarrahi, Meenakshi Ahluwalia, Valdemar Antonio Paffaro, Vincenzo Costigliola, Jack C. Yu, David C. Hess, Krishnan M. Dhandapani, Babak Baban

**Affiliations:** ^1^ Department of Oral Biology and Diagnostic Sciences Dental College of Georgia, University Augusta GA USA; ^2^ Center for Excellence in Research Scholarship and Innovation Dental College of Georgia Augusta University Augusta GA USA; ^3^ Department of Neurosurgery Medical College of Georgia Augusta University Augusta GA USA; ^4^ Department of Pathology Medical College of Georgia Augusta University Augusta GA USA; ^5^ Department for Cell and Developmental Biology Institute of Biomedical Sciences –Federal University of Alfenas Alfenas Brazil; ^6^ European Medical Association (EMA) Brussels Belgium; ^7^ Children's Hospital of Georgia and Medical College of Georgia Augusta University Augusta GA USA; ^8^ Department of Neurology Medical College of Georgia Augusta University Augusta GA USA

**Keywords:** apelin, ARDS, cannabidiol, CBD, COVID‐19, inflammation

## Abstract

Considering lack of target‐specific antiviral treatment and vaccination for COVID‐19, it is absolutely exigent to have an effective therapeutic modality to reduce hospitalization and mortality rate as well as to improve COVID‐19‐infected patient outcomes. In a follow‐up study to our recent findings indicating the potential of Cannabidiol (CBD) in the treatment of acute respiratory distress syndrome (ARDS), here we show for the first time that CBD may ameliorate the symptoms of ARDS through up‐regulation of apelin, a peptide with significant role in the central and peripheral regulation of immunity, CNS, metabolic and cardiovascular system. By administering intranasal Poly (I:C), a synthetic viral dsRNA, while we were able to mimic the symptoms of ARDS in a murine model, interestingly, there was a significant decrease in the expression of apelin in both blood and lung tissues. CBD treatment was able to reverse the symptoms of ARDS towards a normal level. Importantly, CBD treatment increased the apelin expression significantly, suggesting a potential crosstalk between apelinergic system and CBD may be the therapeutic target in the treatment of inflammatory diseases such as COVID‐19 and many other pathologic conditions.

## 1 INTRODUCTION

COVID‐19 pandemic has profoundly affected human life, inducing high patient morbidity and mortality while stressing health care systems worldwide. SARS‐CoV‐2, the highly infectious agent responsible for the COVID‐19 pandemic, is a novel coronavirus that utilizes a glycosylated spike protein to enter human cells via the angiotensin‐converting enzyme 2 (ACE2) receptor. The lung is a primary site of entry for SARS‐CoV‐2, as evidenced by massive pulmonary inflammation and development of acute respiratory distress syndrome (ARDS).^1^ These changes frequently result in a cytokine storm that contributes to diffuse alveolar damage, alveolar capillary leakage, severe hypoxaemia, intense pulmonary oedema and pulmonary fibrosis.^2^

Cannabidiol (CBD) is a non‐psychotropic phytocannabinoid that regulates immune responses in multiple experimental disease models, including work by our laboratory showing a benefit following ARDS‐like injury in mice.^3^ Consistent with our findings, a recent commentary, based on anecdotal reports, supports the therapeutic use of CBD in COVID‐19‐infected patients.^4^ While a number of mechanisms are postulated to mediate the anti‐viral benefits of CBD, including down‐regulation SARS‐CoV‐2 receptors in human epithelia and suppression of pro‐inflammatory cytokine production (eg interleukin‐1β (IL‐1β), IL‐6, tumour necrosis factor‐α (TNF‐α), chemokine (CC‐motif) ligand 2 (CCL2), chemokine (CC‐motif) ligand 3 (CCL3)), this issue remains largely unresolved and, once elucidated, may identify novel approaches to improve outcomes in COVID‐19‐infected patients.^5^

Apelin, an endogenous, multi‐functional ligand for the G protein‐coupled receptor, APJ, also serves as a second catalytic substrate for ACE2.^6^ Apelin is generated from a 77‐amino acid precursor and undergoes proteolytic cleavage to generate biological active fragments, including apelin‐36, apelin‐19 and apelin‐13. An endogenous protective role was postulated for activation of the apelin/APJ axis (Apelinergic system) after lung injury, via proposed mechanisms including suppression of the immune activating transcription factor, NF‐κB and inhibition of innate immune infiltration/activation via attenuated expression of CCL2, CCL3, CCL4, CCL7 and TNF‐α.^7^ Of interest, both apelin and APJ are widely expressed throughout the lung, heart, liver, gut, kidney and central nervous system,^8^ spatially overlapping expression of the endocannabinoid system while interaction between the endocannabinoid system and apelin limits liver fibrosis.^9^ Based on these studies, we suggested that regulation of the apelinergic system by CBD may limit excessive pulmonary inflammation after experimental ARDS.

## 2 MATERIALS AND METHODS

Polyinosinic:polycytidylic acid (Poly(I:C)), a high molecular weight, synthetic analog of double stranded RNA (dsRNA), was used to recapitulate the histopathological, physiological and immune features of ARDS associated with SARS‐CoV‐2 infection (As described in our recent publication), including low oxygen saturation, lymphopenia, elevated frequencies of neutrophils/monocytes, excess production of pro‐inflammatory cytokines and destruction of lung morphology.^3^, ^10^ Adult (12 weeks) male C57Bl/6 mice were block randomized into one of three experimental groups (n = 10 mice/group) by a blinded investigator. Group I received intranasal, once daily administration of sterile saline for three consecutive days to serve as a control. Group II received intranasal, once daily administration of Poly I:C (100 µg in 50 µL in sterile saline) for three consecutive days to mimic ARDS. Group III received intranasal, once daily administration of Poly I:C (100 µg in 50 µL in sterile saline) for three consecutive days, with intraperitoneal administration of CBD (isolate CBD, THC‐free, 5 mg/kg body weight, Canabidiol Ltd, Dublin, Ireland), first dose two hours after the second Poly(I:C) treatment and every other day for a total of 3 doses to the treatment group. Blood oxygen saturation was quantified via the carotid arteries using a portable pulse oximeter at study initiation (day 0) and once daily for the duration of the study. Mice were euthanized at study day nine. Blood and lung tissue were harvested and subjected to flow cytometry, immunofluorescence and histological analysis, as detailed previously by our laboratory (3). All flow cytometry data were analysed using the FlowJo V10 software while immunofluorescence, and histological preparations were analysed and imaged by fluorescence and bright field microscopy. As for additional histological evaluation, Masson's Trichrome staining was used for the detection of collagen fibres in lung on formalin‐fixed, paraffin‐embedded sections. The collagen fibres stained in blue and the background is stained red. Sections were examined and analysed using bright field microscopy imaging.

## 3 RESULTS

**Figure 1:**
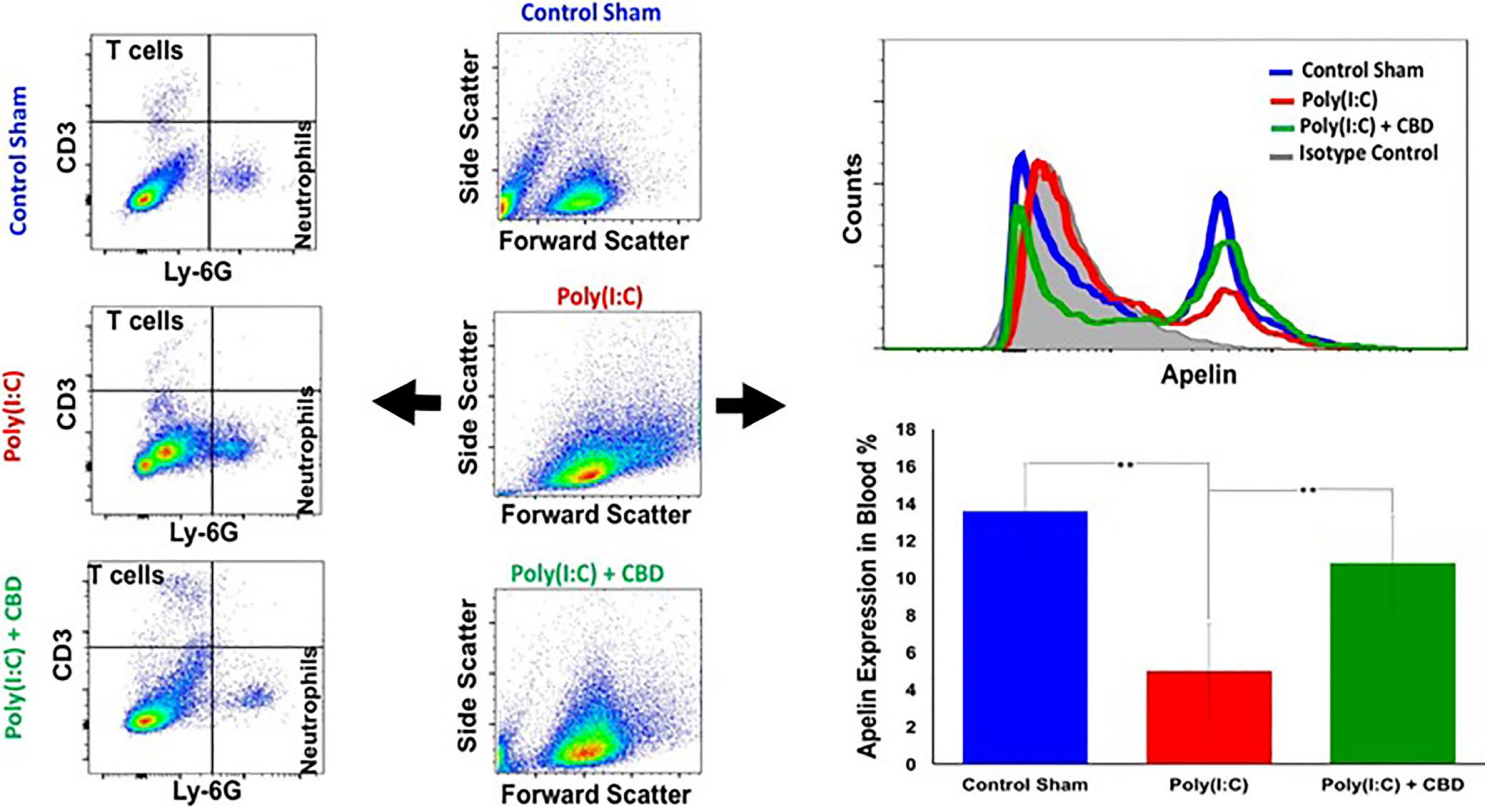
CBD improved the symptoms of Poly(I:C)‐induced ARDS and normalized the expression level of apelin in the blood. Intranasal administration of poly(I:C) demonstrated a reduction in the blood T cells while increased the neutrophils compared with the sham control group (dot plot flow cytometry panels). Further, intranasal poly(I:C) reduced apelin expression in whole blood of mice (Histogram and bargraph panels), as assessed by flow cytometry. Administration of CBD attenuated these effects. The bargraphs are representing the average of values for 10 mice per group (**P < .03)

Flow cytometry analysis of whole blood showed that Poly(I:C)‐treated mice exhibited a pattern of lymphopenia, lower frequency of T cells and elevated rate of neutrophils compared with the sham control group (Figure 1). Further, Poly(I:C)‐treated mice demonstrated significant reduction in the expression level of Apelin compared with the sham control group (Figure 1). Conversely, administration of CBD not only returned decreased T cells and increased neutrophils towards the normal level, but also, enhanced expression of apelin in the blood following poly I:C treatment (Figure 1).

**Figure 2:**
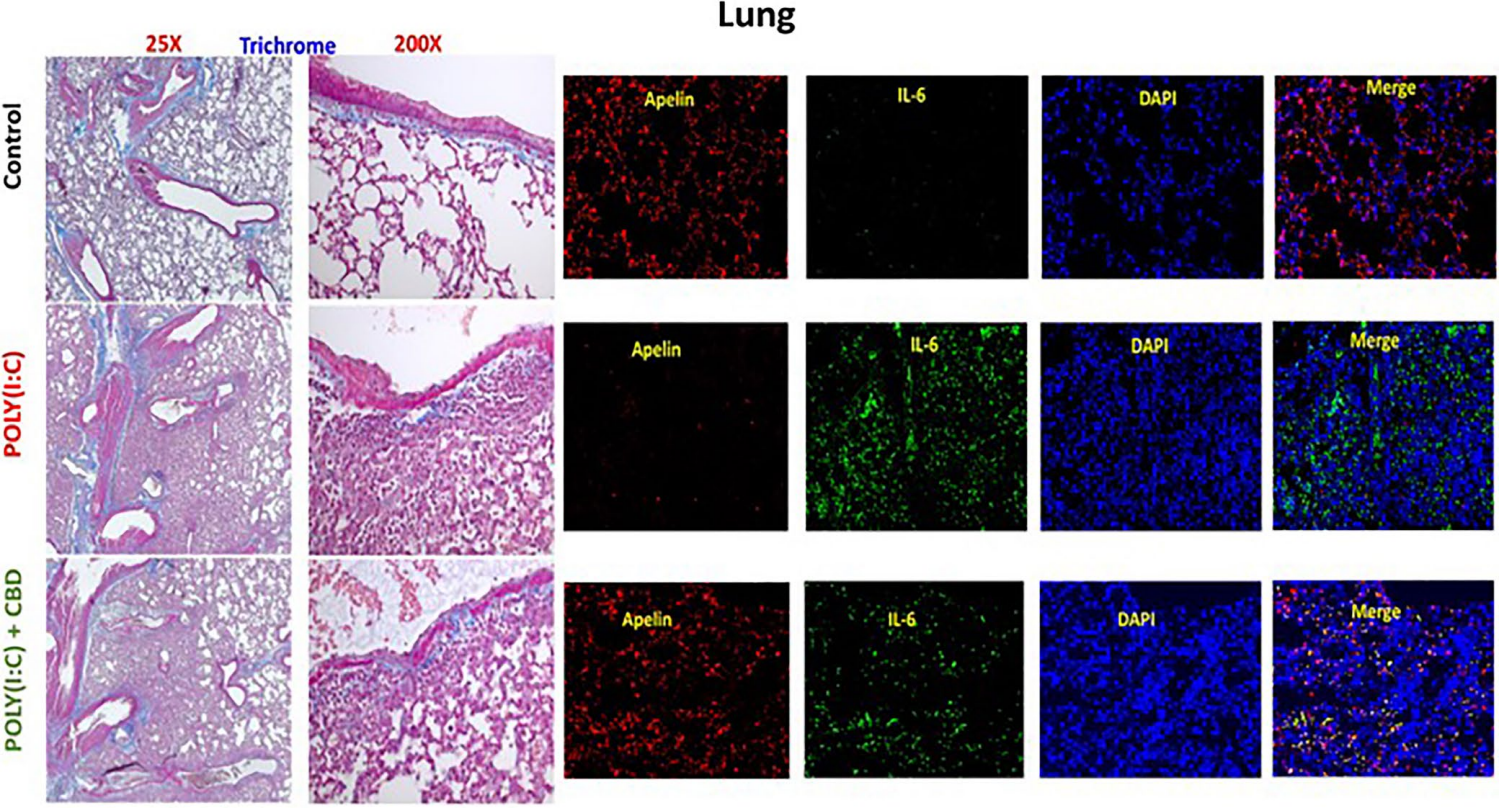
CBD improved the symptoms of Poly(I:C)‐induced ARDS and normalized the apelin expression in the lung tissues. Masson's trichrome analysis of lung tissues showed that intranasal administration of high dose Poly(I:C) caused the destruction of normal morphology and structure of lung, hypertrophy, fibrosis and pulmonary oedema, as compared to the tissues from control group. CBD treatment improved the structure towards the normal architecture (bright filed images on the left panel). Further, immunofluorescence analysis of lung tissue (panels on the right side) showed a decrease in Apelin in Poly(I:C) treated lung compared to normal tissues. CBD treatment normalized the Apelin expression in the lung, indicating the potential protective effect of CBD

In addition, histological examination of lung tissues demonstrated that Poly(I:C) caused significant perivascular and peri‐bronchiolar interstitial inflammatory infiltrate, fibrosis, hypertrophy and pulmonary oedema, as evidenced by the widened interstitial space surrounding the airways and vasculature (Figure 2). The pathological features of poly I:C administration were completely or partially abolished by following administration of CBD (Figure 2). Immunofluorescence analysis of lung tissue revealed a reduction in apelin immunoreactivity after poly I:C treatment, as compared to control mice (Figure 2). Importantly, treatment with CBD increased apelin expression towards control levels in the lung following poly I:C administration (Figure 2).

## 4 DISCUSSION

Our data demonstrate that CBD improves lung structure and exerts a potent anti‐inflammatory effect following experimental ARDS. The beneficial effects of CBD were correlated with the regulation of apelin, an endogenous peptide with protective effects in pulmonary tissue. Thus, apelin may represent a novel molecular target underlying the protective effects of endocannabinoid signalling, including regulation by CBD. Additionally, apelin may represent an unexplored biomarker for the early diagnosis of ARDS. Towards this end, apelin may have utility as a prognostic and predictive biomarker to categorize the risk of deterioration and disease progression. Of similar importance, apelin may serve a powerful and sensitive role as a pharmacodynamic biomarker, providing a biological readout to monitor the efficacy of a therapeutic intervention. Given that apelin is also a substrate for ACE2, these findings may be particularly relevant to the management of COVID‐19. Future pre‐clinical and clinical studies will explore these exciting possibilities.

## DISCLOSURE

All authors have no conflict of interest to declare.

## AUTHOR CONTRIBUTION

**Évila Lopes Salles:** Data curation (equal); Investigation (equal); Methodology (equal); Project administration (equal); Software (equal); Visualization (equal); Writing‐review & editing (equal). **Hesam Khodadadi:** Data curation (equal); Formal analysis (equal); Investigation (equal); Methodology (equal); Software (equal); Supervision (equal); Validation (equal); Visualization (equal); Writing‐review & editing (equal). **Abbas Jarrahi:** Data curation (equal); Software (equal). **meenakshi Ahluwalia:** Data curation (equal); Methodology (equal); Writing‐review & editing (equal). **Valdemar Antonio Jr Paffaro:** Conceptualization (equal); Writing‐review & editing (equal). **Vincenzo Costigliola:** Methodology (equal); Writing‐review & editing (equal). **Jack C Yu:** Conceptualization (equal); Formal analysis (equal); Methodology (equal); Validation (equal); Writing‐review & editing (equal). **David C Hess:** Investigation (equal); Project administration (equal); Supervision (equal); Writing‐review & editing (equal). **Krishnan M Dhandapani:** Conceptualization (equal); Data curation (equal); Funding acquisition (equal); Investigation (equal); Methodology (equal); Project administration (equal); Resources (equal); Writing‐review & editing (equal). **Babak Baban:** Conceptualization (lead); Data curation (equal); Formal analysis (lead); Funding acquisition (equal); Investigation (lead); Methodology (lead); Project administration (lead); Resources (equal); Supervision (lead); Validation (lead); Writing‐original draft (lead); Writing‐review & editing (equal).

## Data Availability

The data that support the findings of this study are available from the corresponding author (BB) upon request.

## References

[1] Saxena SK , Kumar S , Maurya VK , Sharma R , Dandu HR , Bhatt MLB . Current insight into the novel coronavirus disease 2019 (COVID‐19). Coronavirus Dis. 2020;2019:1‐8.

[2] Villar J , Zhang H , Slutsky AS . Lung repair and regeneration in ARDS: Role of PECAM1 and Wnt Signaling. Chest. 2019;155:587‐594. 30392791 10.1016/j.chest.2018.10.022 PMC6435939

[3] Khodadadi H , Lopes Salles ÉDS , Jarrahi A , et al. Cannabidiol (CBD) modulates cytokine storm in Acute Respiratory Distress Syndrome induced by simulated viral infection using synthetic RNA. J Cannabis Cannabinoid Res, 2020, July 8 2020.5(3):10‐13. 10.1089/can.2020.0043 PMC7480719 32923657

[4] Esposito G , Pesce M , Seguella L , et al. The Potential of Cannabidiol in the COVID‐19 Pandemic: A Hypothesis Letter. Br J Pharmacol. 2020;10(10):15157 10.1111/bph.15157 PMC7300643 32519753

[5] Nichols JM , Kaplan BLF . Immune Responses Regulated by Cannabidiol. Cannabis Cannabinoid Res. 2020;5(1):12‐31. 10.1089/can.2018.0073 32322673 PMC7173676

[6] Chen LJ , Xu R , Yu HM , Chang Q , Zhong JC . The ACE2/apelin signaling, microRNAs, and hypertension. Int J Hypertens. 2015;2015:5‐10. 10.1155/2015/896861 PMC4359877 25815211

[7] Huang S , Chen L , Lu L , Li L . The apelin‐APJ axis: A novel potential therapeutic target for organ fibrosis. Clin Chim Acta. 2016;456:81‐88. 26944568 10.1016/j.cca.2016.02.025

[8] Kawamata Y , Habata Y , Fukusumi S , et al. Molecular properties of apelin: Tissue distribution and receptor binding. Biochim Biophys Acta. 2001;1538(2–3):162‐171. 11336787 10.1016/s0167-4889(00)00143-9

[9] Melgar‐Lesmes P , Perramon M , Jiménez W . Roles of the Hepatic Endocannabinoid and Apelin Systems in the Pathogenesis of Liver Fibrosis. Cells. 2019;8:1311. 10.3390/cells8111311 PMC6912778 31653030

[10] Stowell NC , Seideman J , Raymond HA , et al. Long‐term activation of TLR3 by poly(I:C) induces inflammation and impairs lung function in mice. Respir Res. 2009;1(10):43. 10.1186/1465-9921-10-43 PMC2694181 19486528

